# Study on the Role of the Common House Fly, *Musca domestica*, in the Spread of ORF Virus (Poxviridae) DNA under Laboratory Conditions

**DOI:** 10.3390/microorganisms9112185

**Published:** 2021-10-20

**Authors:** Donato Antonio Raele, John G. Stoffolano, Ilaria Vasco, Germana Pennuzzi, Maria Concetta Nardella La Porta, Maria Assunta Cafiero

**Affiliations:** 1 Istituto Zooprofilattico Sperimentale della Puglia e della Basilicata, Via Manfredonia 20, 71100 Foggia, Italy; ilaria.vasco@izspb.it (I.V.); germana.pennuzzi@izspb.it (G.P.); concetta.nardella@izspb.it (M.C.N.L.P.); mariaassunta.cafiero@izspb.it (M.A.C.); 2 Stockbridge School of Agriculture, University of Massachusetts, Amherst, MA 01003, USA; stoff@umass.edu

**Keywords:** ORF virus (sore mouth), housefly, crop, regurgitation, vectorial role

## Abstract

ORF virus (Poxviridae) is the causative agent of contagious ecthyma (soremouth), a disease primarily affecting sheep and goats worldwide, but also humans exposed to disease-ridden animals. Pathogens are shed with scabs, and infection mainly occurs by direct contact. Although the disease is relatively benign and self-limiting, the morbidity rate is high in livestock with subsequent significant financial and economic impact. The aim of the study was to experimentally investigate the potential for the housefly, *Musca domestica*, to act as a mechanical vector of the virus. Homogenate of crusted scabs from ORFV-positive sheep (Italy, Apulia) were used to infect laboratory-reared flies. Flies walking on viral mixture and flies inoculated on their wings were individually placed in Falcon tubes and the ORFV DNA was searched by PCR on tube walls; flies were fed on the same homogenized crusts and their crop and spots (vomit and feces) molecularly examined for ORF DNA at 2, 4, and 6 h. All of the flies (100%) used in the experiments were able to pick up and transmit the viral genome to contact surfaces; 60% were found ORF virus (DNA)-positive in both spots and crop. These results suggest that *M. domestica* could play a role as potential mechanical vector and/or reservoir in the epidemiology of the ORF virus infection. Thus, house fly management should be considered in the measures to control the disease in ovine–caprine farms.

## 1. Introduction

ORF virus (ORFV), the prototypical species of parapoxviruses (Poxviridae), is an epitheliotropic DNA-virus causative agent of the contagious ecthyma (EC), a worldwide pathology primarily affecting sheep and goat farming, but also affecting wild ungulates [1]. Occasionally, infection can occur in humans, mostly in subjects exposed occupationally to animal-infected sources, such as farmers, breeders, shearers and veterinarians [2]. Spread of the infection commonly occurs through direct contact between sick to healthy subjects or through fomites, including thorny plants. In susceptible animals, ORFV enters its hosts through small continuous skin solutions and usually remains confined to the epidermis inducing the formation of papules, nodules or vesicles that develop in thick crusts or heavy scabs mainly on the lips, nostrils, eyes, breast and distal part of the limbs [3]; high fever and mortality are observed in severe forms, while moderate symptomatology occurs in reinfection [1]. Humans typically develop a single, painful nodule on the exposed areas, mostly hands and arms and, occasionally, the face [4]; more rarely, children are affected [5]. According to the 2001 survey of the National Animal Health Monitoring System (NAHMS) on behalf of the United States Department of Agriculture Animal and Plant Health Inspection Service, 40% of operations carried out on US sheep showed the presence of cases of ORFV infection in these animals in the period 1997–2000; the incidence of occupational human cases reflected the prevalence of infection in sheep and goat populations. ORFV infection is the principal zoonotic viral infection reported by the Public Health Laboratory in the United Kingdom [6]. More recently, human cases have been diagnosed in various countries due to the slaughter of infected animals during events such as the Muslim tradition, the Feast of Sacrifice, as has been reported in Italy [7]. The house fly, *Musca (M.) domestica*, is a classic synanthropic species closely linked to human populations and their activities, including animal husbandry [8]. It can act as a vector for a range of parasitic, bacterial, viral and zoonotic pathogens [9,10,11,12], and there is clear evidence that shows the dipteran can also carry zoonotic dermatophytes such as *Microsporum canis* [13]. Pathogens are transmitted mechanically or via feces and/or regurgitation, including viruses such as poliovirus, coxsackievirus, enteroviruses and influenza virus [9,10,14,15,16]; more recently, Lumpy Skin Disease (LSD) (Poxviridae) virus DNA was also detected in *M. domestica* (Diptera: Muscidae) flies collected during an outbreak in Russia [17]. In Italy, the presence of *M. domestica* has long been documented in cattle, more abundantly in anatomical areas moistened by secretions (muzzle, eyes, nostrils, breast, etc.) [11,12]. The same behavior has been observed in Italian ORFV-positive sheep, where house flies have been seen aggregating on the same body sites which have been shown to be affected by skin lesions when ORFV infection occurs [18,19]. Furthermore, the report of Webbs et al., 1980 [20] suggests that flies should be reassessed as vectors in the epidemiology of sheeppox and goatpox virus (Capripoxvirus), a very similar affliction to ORFV. Based on these observations and the evidence that no data are available in the literature regarding whether the housefly, *M. domestica*, carries ORFV on its body surfaces and/or internal organs and thus has a role in the spread of the virus, we investigated the potential role of this dipteran in ORFV mechanical transmission under experimental conditions.

## 2. Materials and Methods

### 2.1. House Fly Colony

Adult flies were caught from an ovine farm (Foggia, South Italy) using an insect net and rapidly transferred to the laboratory in a box. The specimens were chilled at 4 °C for 5 min, morphologically identified as *M. domestica,* and introduced into a new cage for breeding. After eggs were laid, the emerging larvae were randomly selected and confirmed to be free of ORFV by using molecular analysis [21]. At the same time, the adults used to start the 1st generation were destroyed and the subsequent larvae matured in a wooden framed cage, measuring 95 × 95 × 46 cm, covered on all sides with mosquito netting, a metal bottom, a hole, and cotton sleeves on one side for hand insertion (INFIS 2000, Italy). Flies were kept at temperatures ranging between 20 and 25 °C at 65% relative humidity and a constant photoperiod (12:12 light:dark). The pabulum for larval development consisted of a mixture of bran (300 g), sterilized dry dog food (200 g), brewer’s yeast (7 g) and whole milk (30 mL). Adult flies were fed ad libitum using 46% powdered milk blend, 46% granulated sugar and 8% powered eggs.

### 2.2. Virus and Polymerase Chain Reaction

The virus (starter) was obtained from buccal crusts of sheep from a farm in southern Italy and naturally infected with ORF virus; diagnosis was previously achieved using Electron Microscopy, cell culture, and molecular analyses, as reported in the study by Galante et al., 2019 [18]. Subsequently, the material was stored at −80 °C (collection IZSPB8598PZ, 2013). For the tests, the material was homogenized, and viral DNA was extracted using a commercial kit (GeneJET viral DNA and RNA Purification Kit, Thermoscientific, Waltham, MA, USA), and then, it was used as a template for all tests. Two PCR protocols for the amplification of ORF virus DNA were carried out. The first assay, following the procedure of Kottaridi et al., 2006 [21], was applied to prove the presence or absence of target. As reported by the authors, the endo-point PCR showed a sensitivity up to 0.1TCID 50/mL of the virus stock. This assay uses the 045 ORF fragment as the target gene (isolate OV-SA00, accession number AY186732) [22], which encodes the transcription factor VLTF-. PCRs were performed with a mixture that had a final volume of 50 µL, including 25 µL of RED Taq Ready Mix PCR Reaction Mix (Sigma-Aldrich, St. Louis, MO, USA), 1 mM of each primer and 5 mL of extracted DNA. The cycle of amplification involved an initial denaturation at 95 °C for 5 min and then 40 thermal cycles, consisting of 95 °C for 15 s, 57.7 °C for 30 s and 72 °C for 50 s plus a final extension at 72 °C for 10 min. Amplification products were loaded for electrophoresis on a 2% agarose gel made with Syber^®^Safe (Life Technologies, Carlsbad, CA, USA) and viewed with the Molecular Imager DocTM XR + (Bio-Rad, Berkeley, CA, USA) transilluminator. Amplicons were subsequently purified using the commercial GeneJet PCR purification kit and sequenced using the Sanger method (Big Dye Terminator Kit, Life Technologies, Milan, Italy) using external facilities (Eurofins Genomics Italy, Milan, Italy). All sequences were compared with those present in GenBank using Blast to confirm the identification.

The starter template was analyzed by a second PCR assay. Amplification and quantification of the ORF virus were carried out based on the principle of Syber Green technology using the assay described by Kottaridi et al., 2006 [21], modified in Syber Green for real-time PCR. The standard reaction curve was calculated using the analysis of logarithmic dilutions of a plasmid vector containing a copy of the target sequence (gene target is the 045 ORF fragment encoding the transcription factor VLTF-1). The standard curve of the real-time PCR reaction was generated on the StepOne Real Time PCR device (Applied Biosystem, Bedford, MA, USA) using serial base 10 dilutions of the plasmid recombinant with known concentration obtained previously. Such dilutions (1 × 10^9^ to 1 × 10^−1^ copies/µL) were tested in triplicate and used as a quantitation standard to construct the curve resulting from the detection in reaction of the number of copies of the plasmid and the value of the corresponding threshold cycle (copy target). The detection limit of the reaction was determined based on the highest plasmid dilution that could be amplified (originating a fluorescent signal) with good reproducibility. It was found to be 10 copies/µL of the ORF genome. The melting curve analysis was performed with continuous fluorescence reading between 65 °C and 95 °C. The amplification and melting curve analysis of the PCR products from ORF virus DNA showed a melting temperature in a range of 88.22 °C and 87.92 °C.

### 2.3. Infection of Flies with ORF Virus

A total of three tests were conducted to determine the potential of house flies to harbor and transfer ORFV. For each test, third-generation *M. domestica* adults were used. Experiments were conducted in a Biosafety Level 3 (BL3) laboratory.

Test 1: Efficiency of housefly legs in ORF virus acquisition and dislodgment.

Flies used: 5.

Positive control: ORF virus DNA from ovine scrabs (10^6,4^ copy target).

Negative control: ORF virus-free *M. domestica.*

**Figure 1:**
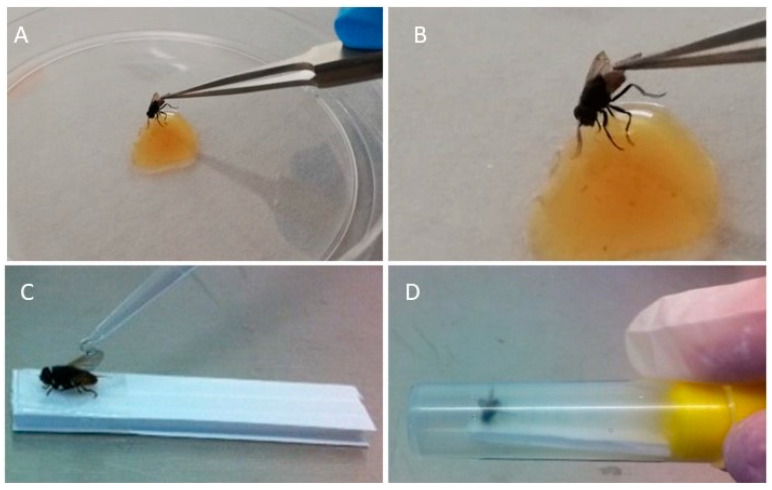
Experiments 1–2: ORFV (from homogenized ovine crusts) contamination of housefly legs (**A**,**B**) and wings (**C**); housefly during flight motion after wing contamination (**D**).

About 80 µL of homogenized ovine crusts was distributed in a Petri dish. Each fly was taken from the breeding cage using a sterile 50 mL Falcon Conical Centrifuge tube and was placed at −20 °C for 1 min to immobilize the dipteran. Subsequently, the specimen was held by the wings using sterilized anatomical tweezers, extracted from the Falcon tube, placed in a Petri dish and observed until it started to recover its natural mobility. At this point, each insect was suspended for 30 s in the drop of homogenate and subsequently transferred to an empty sterile test tube, leaving it free to move along the walls of the tube for 10 min at room temperature (Figure 1A,B). Each fly was euthanized by placing the test tube at 20 °C for 10 min and then removed. Subsequently, one thousand microliters of PBS were added to the test tube, vortexed for 20 s, and the final solution was analyzed to detect the presence or absence of DNA of ORF virus using a PCR, in accordance with Kottaridi et al., 2006 [21].

Test 2: ORFV dislodgment from contaminated wings of house fly during simulated flight motion.

Flies used: 5.

Positive control: ORF virus DNA from crusts (10^6,4^ copy target).

Negative control: ORF virus-free *M. domestica.*

Each specimen of *M. domestica* was collected from the breeding cage using a sterile 50 mL Falcon Conical Centrifuge tube and chilled at −20 °C for 1–2 s. The specimen was held by the wing using fine forceps and immobilized by gluing its legs onto a white plastic strip of 2 × 0.8 cm. About 20 µL of homogenate of ovine crusts was spread onto the upper side of the wings using a micropipette (Figure 1C). Subsequently, the plastic strip was inserted into a sterile 5 mL tube and the fly was left to freely move its wings for 3 min (Figure 1D). The tube was frozen at −20 °C for 10 min, and the plastic strip with the glued fly was removed. In the empty tube, sterile distilled water (1 mL) was dispensed and vortexed for 20 s, and the liquid was collected for DNA extraction and ORFV detection (presence/absence) using PCRs, in accordance with Kottaridi et al., 2006 [21].

Test 3: House fly spots (regurgitation/feces) and crop examination for ORFV.

Flies used: 15.

K+ = ORF virus DNA from crusts (10^6,4^ copy target).

K− = spots (vomit/feces) and crop from flies fed with milk/water mixture (ratio 1:1).

House fly spot (vomit/feces) collection.

**Figure 2:**
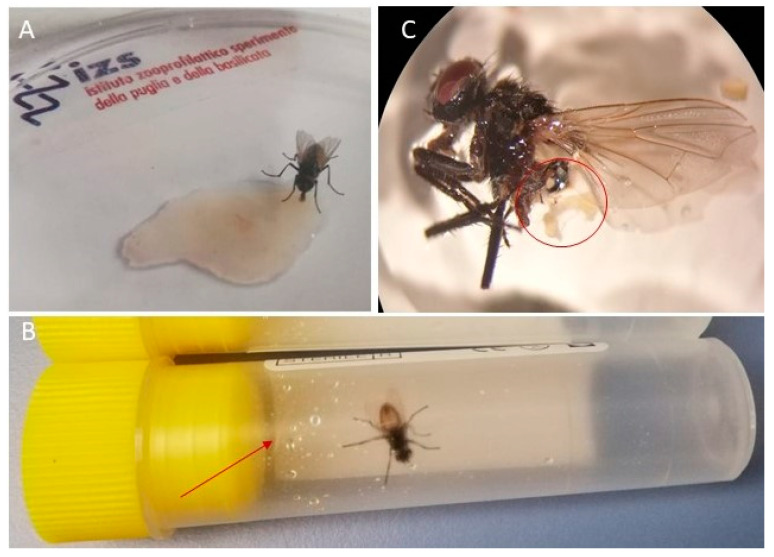
Experiment 3: Housefly contamination by feeding on homogenized ORFV-positive ovine crusts (**A**); housefly spots visible on the walls of the tube test (**B**); dissection of the housefly crop after infective meal (**C**).

The flies fasted for 24 h in the breeding cage were individually collected using a sterile 50 mL Falcon Conical Centrifuge tube. Each fly was chilled at −20 °C for 1–2 s and allowed 30 min of feeding time in a Petri dish where 200 µL of ovine homogenized crusts was previously distributed (Figure 2A). Subsequently, each specimen was chilled at −20 °C for 1–2 s and quickly placed in a sterile 5 mL test tube to regurgitate. A total of 3 test tubes with spots within 2 h, 4 h, and 6 h from the meal was collected from each specimen (Figure 2B). After, each fly was euthanized by freezing, extracted from the test tube, washed 3 times in PBS and placed on a watch glass with a drop of PBS to remove its crop by dissection (Figure 2C). Then, 500 µL of PBS was added in each of the 3 test tubes with individual spots at 2 h, 4 h, and 6 h from the meal and the material collected through a sterile inoculating loop. The liquid was vortexed for few seconds and tested for ORF virus [1], modified in SYBR Green to be assayed in real-time PCR.

### 2.4. Statistical Analysis

Tests 1 and 2: The proportion of flies able to dislodge the virus from wings/legs to contact surfaces after contamination was also estimated at the level of confidence of 0.472 using the Clopper and Pearson procedure.

Test 3: The Friedman test was used to assess whether the virus concentration in the spots (regurgitation/feces) and crops of each tested fly was statistically significant at the three different time points at the level of significance of 0.05.

## 3. Results

### 3.1. Test 1 and Test 2

Test 1: Efficiency of housefly legs in ORF virus acquisition and dislodgment.

Test 2: ORFV dislodgment from contaminated wings of house fly during simulated flight motion.

In each test, ORF virus DNA was detected in the washing liquid collected from each fly specimen (5/5) (100%; amplicons (392 bp) exhibited 100% identity homology with SY17 ORF virus strain (accession number: MG712417.1) All of the flies exposed to the virus (5/5) (100%) were able to acquire the virus on their legs and wings and also to transfer it to contact surfaces (Falcon tube walls). No PCR products were generated in the negative control.

### 3.2. Test 3

Test 3: House fly spots (regurgitation/feces) and crop examination for ORFV.

Out of the 15 tested house flies, 9 (9/15) (60%) fly specimens were found to be ORFV-positive and the remaining (6/15) (40%) were found to be negative. Out of nine flies that were ORFV-positive, four (4/9) flies were positive in both the crop and spots with four (4/9) flies and one only (1/9) positive in spots or crop, respectively. In relation to time, 26% (4/15), 33.3% (11.8%, 61.6%) (5/15) and 40.0% (16.3%, 67.7%) of the infected flies eliminated the virus in spots at the second, fourth and sixth hour from the meal; in 33% (11.8%, 61.6%) (5/15), the virus was detected in the crop, either exclusively or in association with spots (Table 1). By means of the Friedman test, a non-significant difference was found when varying “t” in the three measurements made for spots (1.18, *p* = 0.554). The viral concentration in fly spots (8/9) and in the crop (1/9) was shown to be between 10^3^ and 10^5^ copy target (Ct) values.

## 4. Discussion

The results presented herein provide the first demonstration that *M. domestica* can mechanically carry ORFV (DNA) via both its legs and wings and transfer it to contact surfaces; the dipteran is also able to excrete the ORFV genome by oro-fecal routes (vomit/feces) and to store it in crop. In particular, 100% of the used flies acquired and transferred the ORFV (DNA) to contact surfaces via their legs and wings, while in 60% (9/15) of the fed flies, it was detected in their spots (8/9) (vomit/feces) and crops (1/9) for up to 6 h following the infective food. Our tests confirm that *M. domestica* is structurally able to pick up and mechanically carry ORFV (DNA), such as reported for pathogens; in fact, its external surface is entirely covered by long bristles capable of carrying environmental detritus and pathogens, with over 32 million bacteria carried [23]. All six of their legs have pads capable of secreting and sticking to materials, thus adding the pathogens which can also stick to the pulvillus of fly legs [15]. Our preliminary data also show that flies are able to feed on ORFV (DNA)-positive ovine crusts, dispense the diet into their crop and regurgitate and defecate the viral genome; this suggests that their body surfaces may be contaminated by their infected excreta. Furthermore, ORFV-positive droplets eventually present on the tip of the fly proboscis may be spread by grooming, thus contaminating their legs and possibly their wings, such has been observed for a number of bacteria [24,25]. Evidence shows that grooming increases significantly in flies exposed to bacteria compared to flies not exposed; this suggests that fly cleaning could affect the mechanical transmission of disease bacteria [25] and that a fly’s ability to remove pathogens should also be kept in mind in the spread of ORFV infection. Although tests using live virus are recommended, the results obtained under laboratory conditions appear to be of undoubted interest and strongly suggest for *M. domestica* a role in the epidemiology of ORF virus infection, probably as a mechanical vector in short distances, as already shown for a number of pathogens [26], including viruses [16]. ORF virus was detected in fly spots and crop at least up to the sixth hour with Ct-values between 10^3^ and 10^5^, slightly lower than the starter (10^6,4^). This result is particularly interesting because it further strengthens the importance of the potential role of the common house fly in the mechanical dispersion of contagious ecthyma; however, more studies using live virus to infect flies and subsequent viral isolations are necessary to exclude or to confirm the replication of ORFV in adult fly’s gastrointestinal tract. Frequent meals on infected substrates could be expected to accumulate pathogens in a fly’s alimentary system [15]. The decrease in Ct-value in flies that tested positive could be not only linked to the capability of each fly to take a different quantity of food but also to the distribution of the ORFV (DNA) in each of the 15 drops (200 mL each) of food administered to each of the 15 specimens. The percentage of flies (6/15) (40%) that tested negative for the virus after natural feeding may have depended on the greater or lesser intake of food and viruses ingested, with minimal quantities not being detected by the method used. However, the involvement of more flies in the tests would be useful to evaluate the variability of their behavior (e.g., the different amount of ingested food for each fly, etc.).

**Figure 3:**
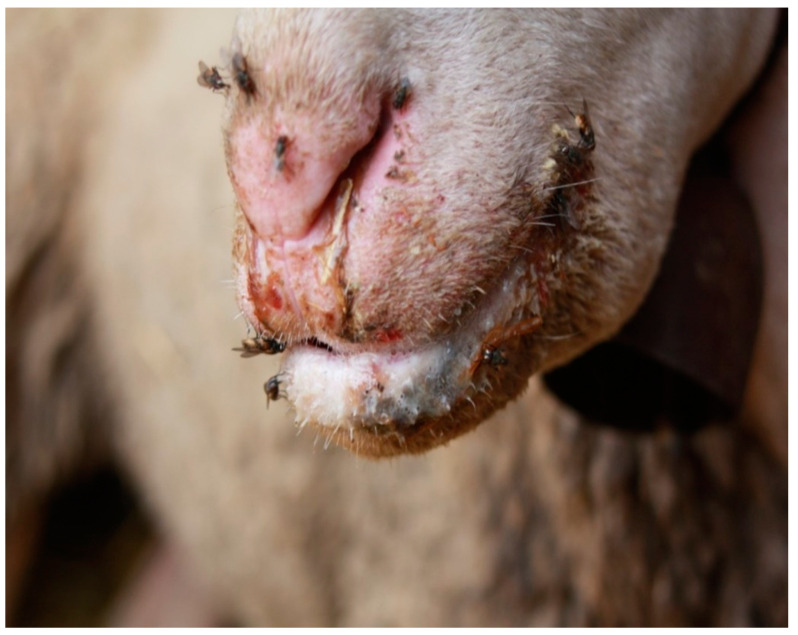
House flies feed on buccal lesions and saliva of ORFV-infected sheep during an outbreak.

Furthermore, the presence of milk receptors on this insect makes it particularly attracted to some anatomical areas, such as the nipples of lactating females and the snout of lambs and kids; such locations are also those where the flies tend to concentrate and/or where ORFV-related lesions begin to develop (Figure 3). For these reasons, flies are more likely to come in contact with the virus and become contaminated; this is also due to the typical walking pattern of flies that involves moving, stopping and returning to the same surface. We also know that the nature and composition of the substrate on which flies feed and/or move may have an influence on contamination [27]; interestingly, our experiments show that homogenized ovine ORFV (DNA)-positive crusts are suitable to infect house flies; this condition is very similar to what could occur in nature during an ORFV outbreak where the fresh scabs of infected animals could represent an important source of the virus for synanthropic flies, such as *M. domestica*. ORFV is very hardy and persists on farm material and in the environment for months to years [28]; for this reason, to really understand the mechanical role of house flies in the dispersion of ORFV infection, studies should be prolonged well after outbreaks to verify the presence of the virus in wild flies collected in the same flock. ORF infection in ovine–caprine farms and in humans tends to occur worldwide during the spring–summer season [29], including in southern Italy where outbreaks peak from June to September [18]; interestingly, in these months, the *M. domestica* population also reaches its highest density. Particular attention should be given to other viral diseases, such as lumpy skin disease in cattle, where discharges from the infected hosts [30] make a ready source of infective fluids available to be consumed, stored in the crop, regurgitated and even defecated. As stated by Sprygin et al., 2019 [17], “*Thus, while low virus titers within nasal or other discharges are indeed likely to lower the risk of contact transmission, there is a need to re-investigate the direct mode of transmission as it pertains to spread of LSDV (lumpy skin disease virus)*”. In that same paper, the authors address insect vectors as potential vectors of LSDV and even make a case that suggests house flies might play a role. As for the practical aspects of the ORF virus, we must focus not only on the economics of this virus [31], but also on its role as an important emerging infectious disease [2,32,33] both in sheep/goat hosts and with respect to its zoonosis and the infection of humans [34]. The persistence of the ORF virus in the environment, as well as in hosts, creates an ever-increasing contact for zoonoses or spillover into the human population. Based on our study, we highly recommend more attention and research be given to non-biting flies, especially adult house flies as mechanical vectors of the ORF virus. In light of the results obtained, this experimentation seems particularly important to use as it represents a model that can also be used to carry out infections with viruses other than the one employed.

### Observations and Limitations of the Study

The study was based on observations related to the behavior of house flies during ORFV outbreaks; consequently, our results can be considered paradigmatic until “in field” studies become available:

- Particularly, crusty lesions in infected sheep/goats represent the preferential site for nourishment of house flies (Figure 3).
- Fresh ORFV-infected crusts, containing live virus, are the main source of environmental contamination.
- Our study identified the presence of ORF viral genome in flies; no information is available on the presence of live virus in the flies.
- More flies should be involved in future studies to better take into consideration the variability of behavior of flies.
- Long-term studies based on sampling of flies in the field during ORF virus outbreaks would be needed to really understand the role of house flies in the spread of the pathogen.

## Figures and Tables

**Table 1:** Experiment 3: Detection of ORFV-DNA using RT-PCR in housefly spots and crops. Fly spots were examined at 2 h, 4 h and 6 h after the infective feed. Ct = copy target.

Flies Infected	Starter (Infected Ovine Crusts)	K−/K+	2 h	4 h	6 h	After 6 h	Positive Flies/Total
			ORFV-DNA in Fly Spots (Vomit/Feces)	ORFV-DNA in Fly Spots (Vomit/Feces)	ORFV-DNA in Fly Spots (Vomit/Feces)	ORFV-DNA in Fly Crop	
Fly 1	*Ct* 10^6,4^	−/+	-	-	-	-	60% (9/15)
Fly 2	*Ct* 10^6,4^	−/+	*Ct* 10^5^	-	*Ct* 10^4^	-	
Fly 3	*Ct* 10^6,4^	−/+	-	-	-	*Ct* 10^5^	
Fly 4	*Ct* 10^6,4^	−/+	-	-	-	-	
Fly 5	*Ct* 10^6,4^	−/+	-	-	-	-	
Fly 6	*Ct* 10^6,4^	−/+	*Ct* 10^4^	*Ct* 10^4^	*Ct* 10^4^	*Ct* 10^5^	
Fly 7	*Ct* 10^6,4^	−/+	-	*Ct* 10^4^	-	-	
Fly 8	*Ct* 10^6,4^	−/+	*Ct* 10^3^	-	-	-	
Fly 9	*Ct* 10^6,4^	−/+	-	*Ct* 10^4^	*Ct* 10^4^	*Ct* 10^5^	
Fly 10	*Ct* 10^6,4^	−/+	-	-	*Ct* 10^4^	*Ct* 10^5^	
Fly 11	*Ct* 10^6,4^	−/+	-	*Ct* 10^4^	*Ct* 10^4^	*Ct* 10^4^	
Fly 12	*Ct* 10^6,4^	−/+	*Ct* 10^3^	*Ct* 10^3^	*Ct* 10^4^	-	
Fly 13	*Ct* 10^6,4^	−/+	-	-	-	-	
Fly 14	*Ct* 10^6,4^	−/+	-	-	-	-	
Fly 15	*Ct* 10^6,4^	−/+	-	-	-	-	
Results (%)			26% (4/15)	33% (5/15)	40% (6/15)	33% (5/15)	

## Data Availability

All relevant data are provided in the manuscript. Raw data can be made available on reasonable demand.
